# Water Treatment Effect, Microbial Community Structure, and Metabolic Characteristics in a Field-Scale Aquaculture Wastewater Treatment System

**DOI:** 10.3389/fmicb.2020.00930

**Published:** 2020-06-05

**Authors:** Zhifei Li, Ermeng Yu, Kai Zhang, Wangbao Gong, Yun Xia, Jingjing Tian, Guangjun Wang, Jun Xie

**Affiliations:** ^1^Key Laboratory of Tropical and Subtropical Fishery Resource Application and Cultivation, Pearl River Fisheries Research Institute, Chinese Academy of Fishery Sciences, Guangzhou, China; ^2^Guangdong Ecological Remediation of Aquaculture Pollution Research Center, Guangzhou, China

**Keywords:** biofilm, denitrification, field-scale aquaculture wastewater treatment system, microbial community, nutrient removal

## Abstract

Avoiding and mitigating the introduction of harmful effluent into the environment must be a key part of intensive industrial aquaculture development in order to minimize pollution impacts. We constructed a novel field-scale aquaculture wastewater treatment system (FAWTS) involving three-stage purification to efficiently remove nutrients from effluent wastewater. However, the mechanisms of nutrient attenuation in the FAWTS are still unclear. Since microbiota play an important role in the treatment of aquatic pollutants, we hypothesized that the different stages of FAWTS may have enriched various nutrient-metabolizing bacteria, with these promoting nutrient attenuation. We therefore tested microbial metabolic activity, microbial composition, and their metabolic potential using Biolog-GN2 microplate culture and high-throughput sequencing of prokaryotic 16S rRNA gene amplicons. Our results showed that the FAWTS displayed high removal efficiencies for chemical oxygen demand (COD, 74.4–91.2%), total nitrogen (TN, 66.9–86.8%), and total phosphorus (TP, 76.2–95.9%). Simultaneously, microbial metabolic activity for various carbon sources was significantly enhanced in FAWTS biofilms. Denitrifying and phosphorus-removing bacteria such as Rhodobacter were enriched in these biofilms, and genes participating in denitrification and the pathway from methylphosphonate to α-D-ribose-1,5-bisphosphate were increased in the biofilm communities. These results imply that the transformed multistep purification system effectively removed N, P, and COD from aquaculture wastewater by enhancing the bacterial communities involved in these processes. This suggests that contamination-free aquaculture is a feasible goal, and that microbial communities are central to pollutant removal.

## Introduction

Pond aquaculture has become a primary means of foodstuff production (Naylor et al., 2000). China is the largest exporting country of aquacultured products, with more than 2.5 million ha of freshwater aquaculture ponds (Lu et al., 2015) producing a total of 22.11 million tons in 2018 (Fisheries Bureau of Agriculture Ministry of China, 2019). Rapid development of pond aquaculture has led to excess wastewater being discharged to natural aquatic ecosystems (Yang et al., 2017). This waste effluent contains massive amounts of nitrogen (N), phosphorus (P), and organic material (Cao and Wang, 2010; Zhang et al., 2015; Dauda et al., 2019), with approximately 1044 Gg total nitrogen (TN) and 173 Gg total phosphorus (TP) discharged annually to lakes, rivers, and oceans (Zhang et al., 2015), resulting in a serious environmental problem and threats to human drinking water (Zhou and Wen, 2004). Therefore, to protect environmental and public health, removing N, P, and organic matter from aquaculture wastewater is of significant importance (Bartoli et al., 2005; Dudley et al., 2016; Hao et al., 2017). Simple, viable, and energy-saving technological facilities for aquaculture wastewater treatment are required for this purpose.

Various treatment approaches have been widely applied to aquaculture wastewater treatment in China, including constructed wetlands (Summerfelt et al., 1999; Lin et al., 2002; Zhong et al., 2011; Zhang et al., 2016), biofilms (De-Schryver and Verstraete, 2009; Li et al., 2012, 2017), hydrophytes (Redding et al., 1997; Li et al., 2007; Zhu et al., 2011), microalgae bioreactors (Gao et al., 2016), ecological ditches (Liu et al., 2010, 2014), and biofilters (Castine et al., 2013). In vast areas of southern China where pond-based aquaculture is common, there are numerous abandoned ditches and ponds that are used mostly for drainage. Since there is a lack of land and pond resources, transforming such ditches and ponds into onsite multistage purification systems can be a highly efficient means of aquaculture wastewater treatment (Pedersen et al., 2008; Jing and Yuan, 2011; Lin et al., 2016). The diverse assemblages in the circulated aquaculture wastewater, including plants, microbes, fauna, and substrates in the multistage purification system facilitate the coexistence of numerous physical, chemical, and biological processes that remove excess nutrients. Biological processes except plant uptake are considered to be the main pathways for pollutant removal (Ma et al., 2019). The performance of biological wastewater treatment systems relies on microbial community compositions and microbial biomass metabolism (LaPara et al., 2001; Flowers et al., 2013), but very little is known about their processing mechanisms in different treatment units. Community microbial structures have been well documented for freshwater systems in recent years as a result of the development of molecular biological technology (Li et al., 2017; Ni et al., 2018). Considering the importance of microbiota on nutrient metabolism in water, we hypothesized that different stages of a multistage purification system likely enriched different nutrient-metabolizing bacteria, which promote nutrient attenuation.

In this study, we transformed abandoned drainage ditches and ponds into a multistep purification system addressing the problem of excessive nutrient discharge at an aquaculture base with an area of 400,000 m^2^ in the southern city of Huizhou, China. We estimated their contribution to N, P, and chemical oxygen demand (COD) removal in the transformed multistep purification system and investigated microbial community compositions and the metabolism of different functional units using Illumina high-throughput sequencing and Biolog GN2 plate culture. Our findings will contribute to the feasibility of contamination-free aquaculture and characterize microbial mechanisms of nutrient metabolism and geochemical cycling in aquaculture wastewater treatment systems.

## Materials and Methods

### Design and Operation of the Field-Scale Aquaculture Wastewater Treatment System

**FIGURE 1:**
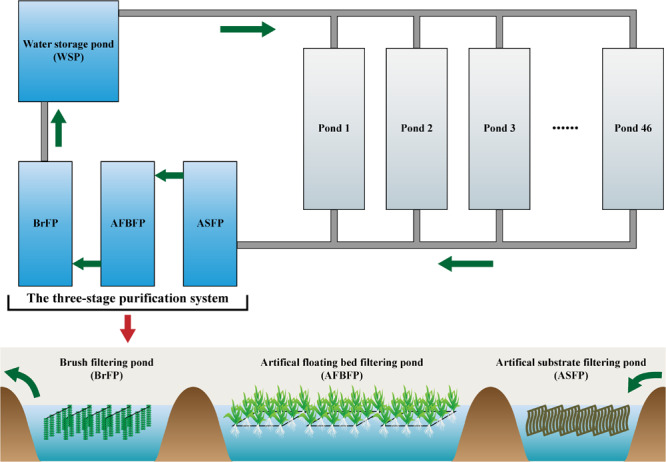
Water treatment process in the field-scale aquaculture wastewater treatment system (FAWTS). Green arrows indicate the direction of water.

A FAWTS was constructed at a Huizhou Caixing Industrial Co., Ltd. aquaculture base located in Huizhou, China (23° 01′ N, 114° 30′ E). The system covered approximately 400,000 m^2^ and consisted of 46 culture ponds of approximately 360,000 m^2^, a three-stage purification system, and a water storage pond of approximately 17,333 m^2^. The culture ponds were used to culture Chinese soft-shelled turtles (*Pelodiscus sinensis*), at a production rate of 2.25 kg/m^2^/year. The turtles were fed a specific manufactured feed (approximately 40% crude protein). The three-stage purification system was revamped from discarded ditches and ponds in 2014, and presently includes an artificial substrate filtering pond (ASFP, approximately 3333 m^2^), an artificial floating bed filtering pond (AFBFP, approximately 20,000 m^2^), and a brush filtering pond (BrFP, approximately 13,333 m^2^; Figure 1). Wastewater was discharged from 46 culture ponds via bottom tubes and flowed through the three-stage purification system. Treated water was then stored and reused for aquaculture.

Artificial substrates, consisting of 5 × 1 m polypropylene non-woven fabric 0.2 cm thick, were hung in the ASFP as our previous report (Li et al., 2017). The actual surface area of each square meter of artificial substratum was 20 m^2^. The density of the artificial substrates, or the ratio of the area of the artificial substrates to superficial area of the artificial substrate filtering pond, was 80%. The rectangular artificial floating beds in the AFBFP was built using 75-mm-diameter polyvinyl chloride (PVC) tubes. Water spinach (*Ipomoea aquatica*) was planted on the artificial floating beds, accounting for 40% of the AFBFP area. Each square meter of the BrFP was hung with eight biological brushes using 1600 acerose polyethylene wires per brush with an effective length of 80 cm. The FAWTS was operated in advance of wastewater treatment for biofilm growth and stabilization beginning in January 2014, using approximately 3% culture pond water discharged to the ASFP per day, and inadequate water for the culture ponds was supplied from the WSP.

### Determination of Water Quality Parameters

After biofilm maturation in the three-stage purification system, water samples were collected from the culture ponds, and outfall was collected from the ASFP, AFBFP, BrFP, and the storage pond every 20 days from May to August 2014, during peak aquacultural production (Supplementary Figure 1). Water samples from each source were collected at 30 cm below the surface, stored at 4°C, and analyzed within 6 h. At the same time, water temperature (WT), dissolved oxygen (DO), pH, total dissolved solids (TDS), oxidation–reduction potential (ORP), and conductivity were measured using a YSI multifunctional water quality analyzer (Pro PLUS 4, YSI, United States).

Approximately 150 mL of each water sample was filtered using Whatman GF/C glass fibers with 0.45 μm pore size (Whatman Inc., Clifton, NJ, United States). Concentrations of NH${}_{4}^{+}$–N, NO_3_–N, and NO${}_{2}^{-}$–N in the filtered water samples were measured using Nessler’s reagent colorimetry, ultraviolet spectrophotometry, and *N*-ethylenediamine colorimetry, respectively. TN, TP, and COD in the unfiltered water subsamples (approximately 500 mL) were measured using alkaline potassium persulfate digestion–UV spectrophotometry, ammonium molybdate spectrophotometry, and potassium dichromate, respectively (Huang, 2000).

### Microbial Community Analysis

#### Sampling of Microbial Communities

The community compositions and metabolic activities of microbiota in different sections of the purification system were investigated using Illumina Miseq high-throughput sequencing and Biolog microplate techniques. Microbial samples were collected from culture pond water, biofilms on the artificial substrates, ASFP outfall water, biofilms on the *I. aquatica* rhizosphere in the AFBFP, AFBFP outfall water, biofilms on the brushes, BrFP, outfall water, and water from the storage pond on August 6, 2014. Each biofilm sample was collected from three subsites on the filter and mixed. Approximately 50 g (wet weight) of the biofilms from each site including the artificial substrate, the *I. aquatica* rhizosphere, and the brush was cut into fragments and placed in 500-mL sterile plastic sampling bottles. Three replicate 1-L samples were collected from each location, totaling 24 samples altogether. These were stored at 4°C and transported to the laboratory for Biolog plate analysis and DNA extraction.

#### Biolog Manipulation and Data Analysis

Microbial metabolic activities from water samples and biofilms were measured using Biolog-GN2 microplates (Biolog Inc., Hayward, CA, United States) (Adedayo et al., 2004). Microplates were warmed to 25°C before samples were added. Water and biofilm samples were diluted using 0.9% physiological saline under sterile conditions. Each 150 μL of treated water sample, or biofilm solution sample containing approximately 10^3^ cells (quantified using plate counts), was added into the microplate wells. These were then covered and incubated at 25°C. The absorbance of each cell was measured using a Multiskan MK3 microplate reader (Thermo Fisher Scientific, Vantaa, Finland) at 590 nm every 24 h and the treated samples were incubated for 9 d. Average absorbance values at 48 h were selected for further analysis, as per Tiquia (2010).

Microbial metabolic activities were measured using average well color development (AWCD), calculated as AWCD = Σ(*C*_i_ – *R*)/95, where *C*_i_ was the absorbance of the 95 wells and *R* was the absorbance of the control well (Garland, 1996). If *C*_i_ was less than *R*, optical density (OD) was set as 0. Carbon sources in the microplate could be classified as amino acids (20), carbohydrates (34), carboxylic acids (26), polymeric compounds (5), or amines (10). AWCD values of plates incubated for 24 h were used to assess microbial metabolic activity of sole-carbon sources (Choi and Dobbs, 1999). Data analysis was conducted according to Tiquia (2010). The maximum AWCD of each carbon source in the water and biofilm samples was treated as 100% to calculate the relative AWCD for the six types of carbon sources.

#### Composition Analysis of Microbial Communities From the FAWTS

Each 150-mL pond water sample was filtered using a 0.22 μm GF/C filter cut into fragments and placed in a 50-mL sterile centrifuge tube for DNA extraction. Each 3-g sample of biofilm was weighed and added to 150 mL of sterile water. The mixture was vortex-mixed eight times for 5 min each, then centrifuged at 20,000 *g* for 15 min at 4°C. Bacterial DNA was extracted from the supernatant using an extraction kit (Omega Scientific, Tarzana, CA, United States) following the manufacturer’s protocol. DNA concentration and purity were evaluated using 1% agarose gels. DNA was diluted to 1 ng μL^–1^ using sterile water prior to further amplification.

The V4 hypervariable region of the 16S rRNA gene was amplified using the 515F and 806R primers with sample-specific barcodes (Yan et al., 2016; Li et al., 2017). Polymerase chain reaction (PCR) was performed in duplicate with a 30-μL reaction mix containing 15 μL of Phusion High-Fidelity PCR Master Mix (New England Biolabs, Ipswich, MA, United States), 0.2 μM of each primer, and approximately 10 ng of template DNA. Thermal cycling conditions were as reported in Li J. et al. (2014). The two PCR products were mixed together, and then the PCR amplicons of all samples were mixed at equal density ratios and purified using the GeneJET Gel Extraction Kit (Thermo Fisher Scientific, Waltham, MA, United States). Sequencing libraries were constructed using the NEB Next Ultra DNA Library Prep Kit for Illumina (New England Biolabs, Ispwich, MA, United States), according to the manufacturer’s instructions, and index codes were added. Finally, libraries were sequenced using the Illumina Miseq platform (Beijing Novogene Corporation, Beijing, China) and 250-bp paired-end reads were generated.

The paired-end reads from the raw DNA fragments were merged using FLASH software. The merged tags were assigned to each sample according to the sample-specific barcodes and chimeric sequences were identified and removed as per Huang et al. (2018), Xiang et al. (2018), and Ni et al. (2019). To eliminate the effect of sequencing depth on subsequent analyses, sequences from each sample were resample according to the minimum number of sequences in the samples (Ni et al., 2017; Huang et al., 2018). Sequences with ≥97% similarity were assigned to the same operational taxonomic unit (OTU) using UPARSE software (Edgar, 2013). Representative sequences of each OTU were used to annotate their taxonomies according to the Ribosomal Database Project (RDP) classifier (Wang et al., 2007). In-house Perl scripts were used to analyze alpha and beta diversity.

Redundancy analysis (RDA), heatmap profiling, non-parametric multivariate analysis of variance (PERMANOVA, Anderson, 2001), and *t*-tests were conducted using R software with the vegan package (Dixon, 2003). STAMP software was used to screen significantly different OTUs and genes in the microbiota among different sections in the FAWTS (Parks et al., 2014). The functional genes of the microbiota were calculated using PICRUSt on the basis of community structures (Langille et al., 2013).

### Data Availability

The sequencing data have been submitted to the National Center for Biotechnology Information (NCBI). Merged Sequence data were deposited in the NCBI Sequence Read Archive (SRA) under accession number PRJNA600293.

## Results

### Purification Performance of the FAWTS

Measurements of COD, TN, TP, NH${}_{4}^{+}$–N, NO${}_{3}^{-}$–N, NO${}_{2}^{-}$–N, and total removal efficiencies (TRE) in the culture ponds and water storage pond over the sampling period indicated that the FAWTS operated steadily, and efficiently maintained a balance of TN, TP, and COD. TREs from the culture ponds to the storage pond of TN, TP, and COD reached 66.9% (Table 1). COD concentration ranged between 44.8 ± 11.2 mg/L in the culture pond and 7.1 ± 4.1 mg/L in the storage pond. Approximately 85.3 ± 5.2% of the total COD was removed during the experiment. At the beginning of sampling, TN was reduced by 66.9% between the culture pond and the storage pond, representing the lowest removal rate; by the conclusion of sampling, TN was reduced by 86.8%. The mean TRE for TP was 88.5 ± 6.5% and ranged between 95.9% and 76.2%. TREs for NH${}_{4}^{+}$–N, NO${}_{2}^{-}$–N, and NO${}_{3}^{-}$–N were 73.7 ± 6.3%, 74.8 ± 6.5%, and 62.6 ± 7.4%, respectively (Table 1). Water in the FAWTS was aerobic during the experiment, with OD > 3.0 mg L^–1^ and oxidation–reduction potential (ORP) between 34.4 and 149.0 mV (Table 1 and Supplementary Table 1).

**TABLE 1:** Time courses of chemical oxygen demand (COD) concentration, total nitrogen (TN), total phosphorus (TP), ammonia nitrogen (NH${}_{4}^{+}$–N), nitrate nitrogen (NO${}_{3}^{-}$–N), nitrite nitrogen (NO${}_{2}^{-}$–N), and total removal efficiencies (TRE; from culture pond to storage pond) in a field-scale aquaculture wastewater treatment system from May 6 to August 6, 2014.

	**Day**	**COD (mg/L)**	**TN (mg/L)**	**TP (mg/L)**	**NH${}_{4}^{+}$–N (mg/L)**	**NO${}_{2}^{-}$–N (mg/L)**	**NO${}_{3}^{-}$–N (mg/L)**
WCP	May 6	34.1	1.75	0.32	0.394	0.136	1.38
	May 21	70.9	3.4	0.34	0.705	0.152	3.07
	June 6	36.9	2.6	0.24	0.412	0.286	4.06
	June 22	50.7	1.8	0.29	0.647	0.216	2.67
	July 6	39.5	2.76	0.39	0.405	0.197	3.57
	July 22	52.1	1.8	0.35	0.691	0.154	1.28
	August 6	29.5	7.4	0.21	0.789	0.257	2.96
OWASFP	May 6	24.5	1.50	0.17	0.426	0.119	1.06
	May 21	50.9	0.98	0.13	0.691	0.146	3.67
	June 6	22.8	2.8	0.26	0.364	0.211	2.69
	June 22	38.9	0.78	0.09	0.523	0.137	3.05
	July 6	36.4	1.7	0.21	0.516	0.124	2.64
	July 22	31.7	1.9	0.33	0.604	0.116	0.99
	August 6	26.9	4.9	0.12	0.602	0.135	2.54
OWAFBFP	May 6	23.7	1.10	0.21	0.317	0.125	1.29
	May 21	63.4	0.66	0.09	0.581	0.121	2.07
	June 6	12.2	1.4	0.19	0.321	0.129	3.19
	June 22	15.4	1.2	0.28	0.529	0.154	2.13
	July 6	14.6	1.09	0.29	0.426	0.139	3.05
	July 22	21.9	1.02	0.24	0.439	0.101	0.89
	August 6	25.7	5.8	0.19	0.355	0.127	1.69
OWBrFP	May 6	17.4	0.97	0.13	0.268	0.067	0.89
	May 21	34.8	0.78	0.09	0.351	0.096	2.49
	June 6	14.7	0.75	0.01	0.269	0.089	1.06
	June 22	11.8	0.98	0.16	0.366	0.103	1.36
	July 6	19.9	0.9	0.15	0.312	0.096	1.69
	July 22	15.9	0.59	0.09	0.248	0.093	0.77
	August 6	15.4	2.1	0.08	0.267	0.154	1.98
WWSP	May 6	3.24	0.58	0.03	0.101	0.025	0.61
	May 21	15.6	0.8	0.01	0.214	0.038	1.24
	June 6	3.24	0.8	0.05	0.159	0.034	1.27
	June 22	13	0.4	0.01	0.151	0.067	1.09
	July 6	4.66	0.72	0.03	0.129	0.045	1.01
	July 22	6.0	0.32	0.03	0.134	0.047	0.64
	August 6	4.11	0.98	0.05	0.116	0.098	0.78
TRE	May 6	90.5	66.9	89.4	74.2	81.6	55.8
	May 21	78	76.5	95.9	69.6	75	59.6
	June 6	91.2	69.2	77.9	61.4	88.1	68.7
	June 22	74.4	77.8	95.2	76.7	70	59.2
	July 6	88.2	73.9	92.6	68.2	77.2	71.7
	July 22	88.4	82.2	92	80.6	69.5	49.8
	August 6	86.1	86.8	76.2	85.3	61.9	73.5

*TRE indicates total removal efficiencies from the culture pond to the storage pond. OWAFBFP, outfall water from the artificial substrate floating bed filtering pond; OWASFP, outfall water from the artificial substrate filtering pond; OWBrFP, outfall water from the brush filtering pond; WCP, water from culture ponds; WWSP, water from the storage pond.*

### Microbial Metabolic Activities in Different Sections of the FAWTS

**FIGURE 2:**
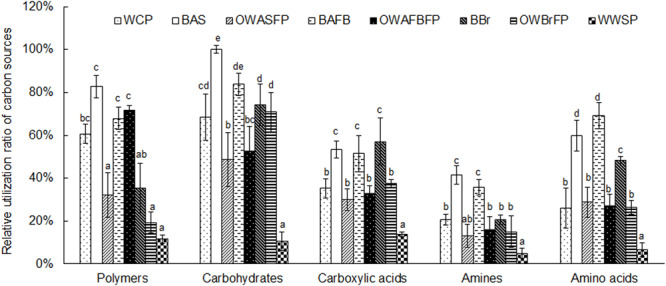
Relative utilization ratio of microbiota from different sections in the field-scale aquaculture wastewater treatment system (FAWTS) to different carbon source types. The highest utilization ratio of carbon sources was treated as 100%, and other carbon sources are shown relative to this standard. OWAFBFP, outfall water from the artificial substrate floating bed filtering pond; OWASFP, outfall water from the artificial substrate filtering pond; OWBrFP, outfall water from the brush filtering pond; WCP, water from culture ponds; WWSP, water from the storage pond. The different lowercase letters indicate significant differences between samples (*P* < 0.05).

Metabolic activities in the biofilms were higher than in the water samples. Among the water samples, activities from culture ponds were higher than in the ASFP, AFBFP, or BrFP. The metabolic activity was lowest in the storage pond water (Figure 2). All measured communities were most efficient at metabolizing carbohydrates and polymers, followed by amino acids and carboxylic acids. Amines were metabolized the slowest (Figure 2). Biofilm communities showed greater utilization efficiency of all carbon sources, implying that the microbiota on the biofilms exhibited a preference for utilizing carbon sources. These results indicate that the biofilms in this system can improve metabolic efficiency.

### Microbial Community Compositions in Different Sections of the FAWTS

**FIGURE 3:**
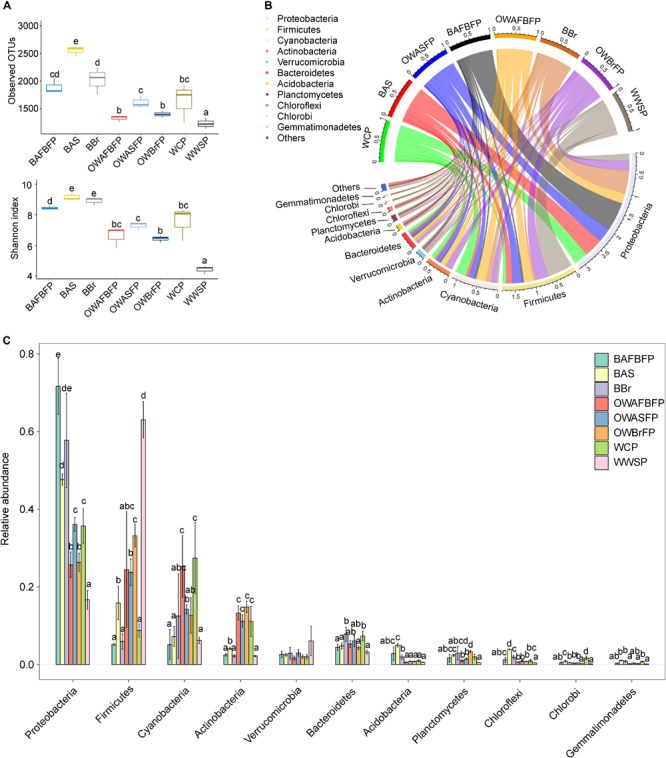
Alpha-diversity indices **(A)** and dominant phylum compositions **(B,C)** of microbiota in the different sections of the field-scale aquaculture wastewater treatment system (FAWTS). BAFBFP, biofilms from the artificial floating bed filtering pond; BAS, biofilms from the artificial substrates; BBr, biofilm from the brush filtering pond; OWAFBFP, outfall water from the artificial substrate floating bed filtering pond; OWASFP, outfall water from the artificial substrate filtering pond; OWBrFP, outfall water from the brush filtering pond; WCP, water from culture ponds; WWSP, water from the storage pond. Different lowercase letters indicate significant differences between samples (*P* < 0.05).

A total of 758,241 high-quality merged sequences were obtained from the 24 samples. To eliminate the effect of sequencing depth on subsequent analyses, 20,112 high-quality sequences were resampled from each sample. All sequences were assigned to 5591 OTUs at 97% similarity. The number of OTUs detected in each sample ranged from 1139 to 2626 (1711.54 ± 440.45). OTU rarefaction curves showed a plateau at 20,000 sequence (Supplementary Figure 2), indicating that this sequencing depth could capture the general structure of the microbial community. There were significant differences in observed OTUs and Shannon indices between different sections of the FAWTS (Figure 3A). Of the obtained sequences, 99.42% were classified into 54 phyla (52 Bacteria and two Archaea). Of those falling within the Bacteria domain, Proteobacteria (39.65 ± 18.08%), Firmicutes (22.51 ± 18.98%), Cyanobacteria (13.82 ± 9.56%), Actinobacteria (7.67 ± 5.38%), Verrucomicrobia (2.85 ± 1.85%), Bacteroidetes (5.41 ± 1.76%), Acidobacteria (1.69 ± 1.57%), Planctomycetes (1.91 ± 1.13%), Chloroflexi (1.34 ± 1.10%), Chlorobi (0.59 ± 0.49%), and Gemmatimonadetes (0.48 ± 0.34%) were regarded as dominant, showing at least one sample with relative abundance >1% (Huang et al., 2018; Ni et al., 2019; Figure 3B). The remaining 2803 sequences (0.58%) could not be taxonomically classified.

The relative abundances of Acidobacteria, Gemmatimonadetes, and Proteobacteria in the biofilms on the brushes, artificial substrate, and *I. aquatica* rhizosphere were significantly higher than those in the water samples, while the relative abundances of Actinobacteria, Cyanobacteria, and Firmicutes in the biofilms were significantly lower, with exceptions for Actinobacteria and Cyanobacteria in the water from the storage pond, and Firmicutes in the water from the culture pond (Figure 3C). A number of bacterial species belonging to the Proteobacteria were denitrifying or dephosphorizing species, implying that the biofilms in the FAWTS enhanced these functions.

**FIGURE 4:**
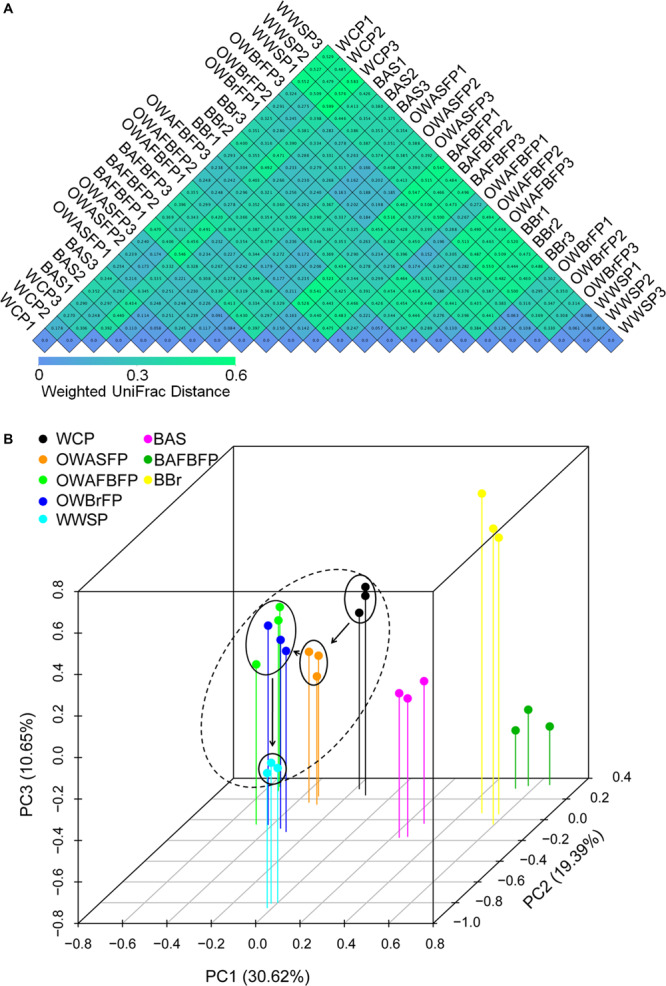
Heatmap showing weighted UniFrac distances **(A)** and redundancy analysis (RDA) profile **(B)** of microbiota OTU compositions in the different sections of the field-scale aquaculture wastewater treatment system (FAWTS). BAFBFP, biofilms from the artificial floating bed filtering pond; BAS, biofilms from the artificial substrates; BBr, biofilm from the brush filtering pond; OWAFBFP, outfall water from the artificial substrate floating bed filtering pond; OWASFP, outfall water from the artificial substrate filtering pond; OWBrFP, outfall water from the brush filtering pond; WCP, water from culture ponds; WWSP, water from the storage pond. Dotted circles indicate microbial samples from water habitats. Solid arrows indicate the direction of water flow in the FAWTS.

Weighted UniFrac distances among the samples collected from the same section were obviously smaller than those between different sections (Figure 4A). An RDA profile drawn based on the microbial OTU compositions showed distinctions between communities in the water samples and those from biofilms (PERMANOVA, *F* = 9.15, *P* = 0.005). Different samples from the same section of the FAWTS were compared and those from different sections were shown to be distinct (PERMANOVA, *F* = 11.02, *P* = 0.005). The microbial compositions of the water of different sections water regularly changed with water flow (Figure 4B).

### Microbial Compositions of Biofilms and Water in Different Sections of the FAWTS

**FIGURE 5:**
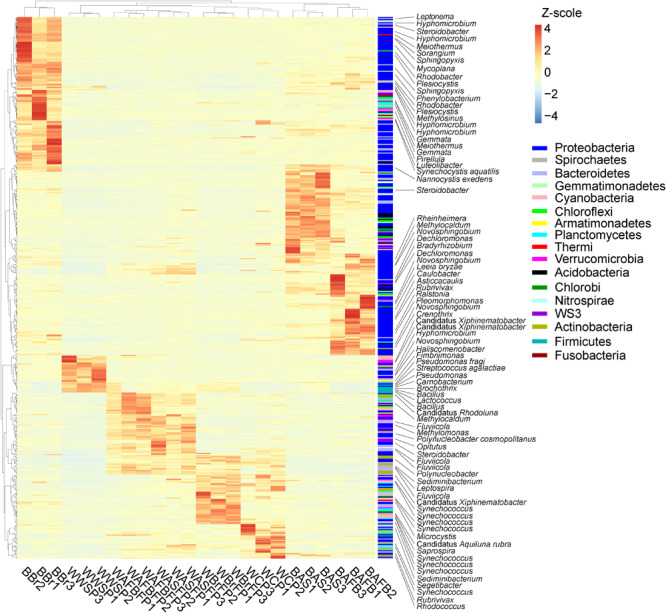
Composition changes in the dominant microbial OTUs in the different sections of the field-scale aquaculture wastewater treatment system (FAWTS). The relative abundances of each OTUs were treated according to the formula log_10_(100 × relative abundance + 1). BAFBFP, biofilms from the artificial floating bed filtering pond; BAS, biofilms from the artificial substrates; BBr, biofilm from the brush filtering pond; OWAFBFP, outfall water from the artificial substrate floating bed filtering pond; OWASFP, outfall water from the artificial substrate filtering pond; OWBrFP, outfall water from the brush filtering pond; WCP, water from culture ponds; WWSP, water from the storage pond. Phylogenetic information for each OTU is shown in Supplementary Table 2.

A total of 503 out of 625 dominant OTUs differed significantly among FAWTS sections or habitats (water or biofilm; Figure 5). In the culture ponds, Proteobacteria were enriched in biofilm communities, and biofilms on artificial substrates showed an increased proportion of bacteria that could not be identified to genus (Figure 5). In water from the storage pond, candidatus *Xiphinematobacter*, *Hyphomicrobium*, *Novosphingobium*, *Haliscomenobacter*, *Fimbriimonas*, *Pseudomonas fragi*, *Streptococcus agalactiae*, *Pseudomonas*, *Carnobacterium*, *Brochothrix*, *Bacillus*, and *Lactococcus* were enriched. In water from the AFBFP, candidatus *Rhodoluna*, *Methylocaldum*, *Fluviicola*, *Methylomonas*, *Polynucleobacter cosmopolitanus*, *Opitutus*, *Steroidobacter*, *Polynucleobacter*, *Sediminibacterium*, *Leptospira*, and *Fluviicola* were enriched. In water from the BrFP, candidatus *Xiphinematobacter*, *Synechococcus*, *Microcystis*, candidatus *Aquiluna rubra*, and *Saprospira* were enriched. In water from the culture pond, *Sediminibacterium*, *Segetibacter*, *Synechococcus*, *Rubrivivax*, and *Rhodococcus* were enriched. In biofilms from the brushes, artificial substrates, and AFBP, the most enriched bacteria, including *Hyphomicrobium*, *Meiothermus*, *Sorangium*, and *Sphingopyxis*, belonged to the Proteobacteria (Figure 5). There was a greater difference in microbiota between biofilms than between water samples from the different treatment sections (Figure 5).

### Changes in Abundance of N and P Metabolic Genes in the Microbiota From Different Sections of the FAWTS

**FIGURE 6:**
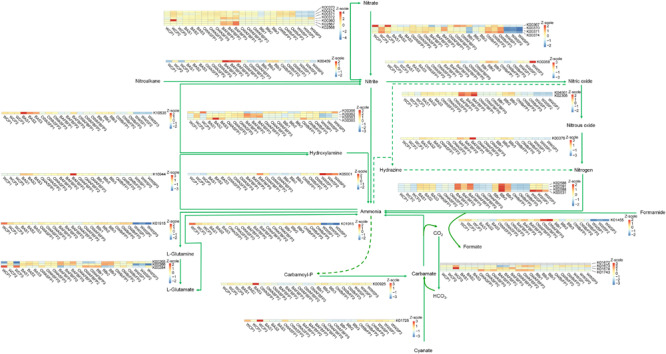
Nitrogen metabolism pathways showing the changes of relative abundance of functional genes in the microbiota from the different sections of the field-scale aquaculture wastewater treatment system (FAWTS). BAFBFP, biofilms from the artificial floating bed filtering pond; BAS, biofilms from the artificial substrates; BBr, biofilm from the brush filtering pond; OWAFBFP, outfall water from the artificial substrate floating bed filtering pond; OWASFP, outfall water from the artificial substrate filtering pond; OWBrFP, outfall water from the brush filtering pond; WCP, water from culture ponds; WWSP, water from the storage pond.

To verify the metabolic enhancement of biofilm microbiota, their functional genes were calculated on the basis of community structure using PICRUSt (Langille et al., 2013) to analyze genes participating in N and P metabolism. Most of the genes participating in denitrification were enriched in the biofilm microbiota (Figure 6). Genes upstream of nitrification (AmoCAB and Hao) were enriched in the biofilm microbiota (Figure 6). In contrast, only one gene participating in anammox was detected, and was significantly increased in the microbiota from the AFB biofilm (Figure 6). This implies that the biofilm microbiota enhanced N removal by increasing populations of bacteria that participate in nitrification and denitrification. For P metabolism, genes participating in the pathway from methylphosphonate to α-D-ribose-1,5-bisphosphate were increased in the biofilm microbiota (Supplementary Table 3).

## Discussion

Aquaculture effluents threaten natural freshwater due to the high concentration of N and P, which can lead to oxygen depletion, eutrophication, and increased turbidity in the receiving waters (Chislock et al., 2013; Penmetsa et al., 2013; Movchan, 2015). In the aquaculture facility studied here, where soft-shelled turtles were raised, feeds consisted of mostly protein, and the cultured animals had a low oxygen requirement. During the culture process, mechanical aeration was reduced. Although DO was higher when sunshine was limited, DO in the turtle ponds was declined to very low (data not shown), which caused low nitrification efficiency and conversion efficiency for COD and NH${}_{4}^{+}$–N. Concentrations of COD, TN, and TP in the culture ponds were higher than have been previously measured in fish culture ponds, implying that wastewater remediation for soft-shelled turtle ponds is more important (Cai et al., 2013). However, emission standards for such facilities are TN < 2.0 mg L^–1^, TP < 0.2 mg L^–1^, and COD < 8 mg L^–1^ (Jiangsu Provincial Bureau of Ocean, and Fisheries, 2011). As such most of the water taken from culture ponds in our study would not meet these standards without treatment. Cai et al. (2013) investigated water quality and volume of effluents from 48 ponds used for culturing crab, shrimp, river prawn, black carp, grass carp, and turtles near Taihu Lake, China, and found that the concentration of pollutants in the effluents often exceeded acceptable limits. Turtle ponds produced the highest gross loads of TN, TP, COD, and total suspended solids. Our study showed that the FAWTS effectively decreased the concentration of pollutants and did not result in pollutant emission during the process of turtle culture (Table 1). The removal efficiencies of TN, TP, and COD were equivalent or even greater than those in previous studies and other domestic wastewater treatment systems (Li Z. et al., 2014; Liu et al., 2016).

Biofilms that effectively promote N, P, and COD removal from freshwater ecosystems have been identified in previous reports (Flemming and Wingender, 2010; Song et al., 2012). A mature biofilm provides a microbial habitat containing both aerobic and anaerobic conditions (Song et al., 2012), with necessary environmental conditions for biological removal of nitrogen by aerobic nitrification, anaerobic denitrification, and anammox. In the aerobic layer, organic materials are degraded and NH${}_{4}^{+}$–N is oxidized to NO${}_{2}^{-}$–N and NO${}_{3}^{-}$–N. In the anaerobic layer, NO${}_{2}^{-}$–N and NO${}_{3}^{-}$–N are transformed to N_2_ (Flemming and Wingender, 2010), or NH${}_{4}^{+}$–N and NO${}_{2}^{-}$–N are directly transformed to N_2_ by anammox bacteria (van Niftrik and Jetten, 2012). Metabolism of P has also been shown to be enhanced by the presence of biofilms in freshwater (Li et al., 2010; Sinsabaugh et al., 2010; Zhao et al., 2012). Our study showed that a field FAWTS can maintain low concentrations of N and P without wastewater discharge during the culture process. The water in the FAWTS was aerobic during the experiment, with OD > 3.0 mg L^–1^ and ORP between 34.4 and 149.0 mV (Supplementary Table 1). This aerobic habitat could provide nitrification conditions, and the high TREs of COD (85.3%) and NH${}_{4}^{+}$–N (73.7%) also implied that the system exhibited high nitration. Simultaneously, the high TREs of NO${}_{2}^{-}$–N (74.7%) and NO${}_{3}^{-}$–N (62.6%) also indicated that the system exhibited high denitrification.

The OTU in the genus *Bacillus* was the most dominant among both water and biofilm microbiota (Figure 5 and Supplementary Table 2). A number of *Bacillus* species are highly effective denitrifiers under both aerobic and anaerobic conditions (Yu et al., 2005; Meng et al., 2009; Lu et al., 2012), and also degrade *N*-acylhomoserine lactone (Defoirdt et al., 2011), and inhibit fish pathogens (Lalloo et al., 2010a, b). Under FAWTS treatment, the relative abundance of this OTU was significantly increased in the water in the storage pond, implying that the treated water was probably more suitable to aquaculture.

Although the OTUs in different phylogenetic clades were increased in different biofilms from different sections of the FAWTS, most of the enhanced phylogenetic clades included denitrification or dephosphorization bacteria (Figure 5 and Supplementary Table 2) and N and P metabolic genes (Figure 6 and Supplementary Table 3). For instance, bacteria belonging to the genus *Rubrivivax* have been reported as decreasing COD (de Lima et al., 2011). Bacteria belonging to family Comamonadaceae can be used in biological phosphorus removal (Ge et al., 2015). Bacteria belonging to the genus *Dechloromonas* can reduce nitrate and anaerobically degrade benzene, toluene, ethylbenzene, and xylene compounds (Coates et al., 2001; Chakraborty et al., 2005). In addition, a number of bacteria in the genus *Novosphingobium* have been reported as degrading organic contaminants such as polychlorophenol (Tiirola et al., 2002), estradiol (Fujii et al., 2003), and carbofuran (Yan et al., 2007). This implies that the biofilms increased removal efficiency of contaminants by enriching functional bacteria.

Bacteria in the genus *Rhodobacter* have also been reported as probiotics (Irianto et al., 2000; Chiu and Liu, 2014). For instance, Chiu and Liu (2014) found dietary administration of the extract of *Rhodobacter sphaeroides* WL-APD911 could enhance the growth performance and innate immune responses of seawater red tilapia. In our study, the relative abundance of *Rhodobacter* was increased in the biofilm microbiota, implying that the FAWTS may help to support the health of aquatic livestock.

## Conclusion

This study provides evidence that a field-scale multistep biofiltration-based aquaculture water self-purification system can efficiently remove N and P from aquaculture wastewater. Biofilm microbiota played an important role in processes that removed N and P. These findings support the feasibility of contamination-free aquaculture and demonstrate the importance of microbiota in pollutant removal.

## Data Availability Statement

The datasets generated for this study can be found in the Merged Sequence data were deposited in the NCBI Sequence Read Archive (SRA) under accession number PRJNA600293. The SRA records can be accessed at https://www.ncbi.nlm.nih.gov/Traces/study/?acc=PRJNA600293.

## Author Contributions

ZL, JX, GW, and EY designed the experiment. ZL, KZ, GW, and WG performed the experiment. ZL, EY, YX, and KZ analyzed the data. ZL, EY, and JT wrote the manuscript.

## Conflict of Interest

The authors declare that the research was conducted in the absence of any commercial or financial relationships that could be construed as a potential conflict of interest.
