# Supplementary material for: Water Treatment Effect, Microbial Community Structure, and Metabolic Characteristics in a Field-Scale Aquaculture Wastewater Treatment System
Source: Front Microbiol. 2020 Jun 5;11:930. doi: 10.3389/fmicb.2020.00930 (PMC7325950; doi:10.3389/fmicb.2020.00930)
Supplement: Supplementary file 1 [file Table_1.docx]

**Table S1.** Physical-chemical characteristics of water in the field-scale aquaculture wastewater treatment system during May 6, 2014 to August 6, 2014.

| Sampling site | T | pH | OD (mg/L) | TDS (mg/L) | ORP (mV) | Conductivity (mS/cm) |
| --- | --- | --- | --- | --- | --- | --- |
| WCP | 27.4.4-33.7 | 8.5-8.8 | 4.3-10.5 | 83.6-176.1 | 34.3-92.3 | 0.139-0.189 |
| OWASFP | 28.3-34.8 | 7.9-8.5 | 4.6-9.4 | 83.2-94.2 | 34.9-74.4 | 0.108-0.128 |
| OWAFBFP | 26.9-34.4 | 7.8-8.4 | 4.2-7.8 | 84.5-88.4 | 40.8-99.1 | 0.101-0.129 |
| OWBrFP | 27.4-33.8 | 7.3-8.1 | 4.0-9.9 | 79.9-96.8 | 42.6-75.9 | 0.099-0.149 |
| WWSP | 26.7-34.0 | 7.8-8.2 | 3.0-8.6 | 37.1-50.1 | 50.8-149.0 | 0.057-0.077 |

WCP: water from culture ponds; OWASFP: outfall water from the artificial substrate filtering pond; OWAFBFP: outfall water from the artificial substrate floating bed filtering pond; OWBrFP: outfall water from the brush filtering pond; WWSP: water from the storage pond.
