# Supplementary figures and images for: Water Treatment Effect, Microbial Community Structure, and Metabolic Characteristics in a Field-Scale Aquaculture Wastewater Treatment System
Source: Front Microbiol. 2020 Jun 5;11:930. doi: 10.3389/fmicb.2020.00930 (PMC7325950; doi:10.3389/fmicb.2020.00930)

**Figure S1** Sampling time array

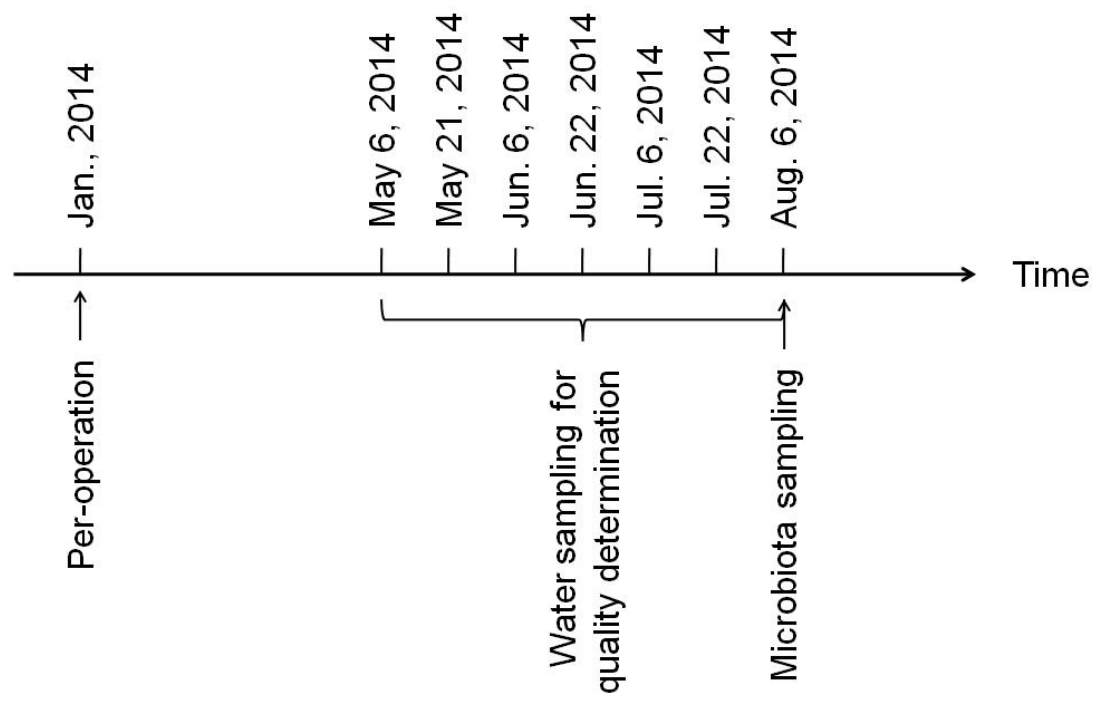

Supplement: Supplementary file 4 [file Image_1.pdf]
