# Supplementary material for: Water Treatment Effect, Microbial Community Structure, and Metabolic Characteristics in a Field-Scale Aquaculture Wastewater Treatment System
Source: Front Microbiol. 2020 Jun 5;11:930. doi: 10.3389/fmicb.2020.00930 (PMC7325950; doi:10.3389/fmicb.2020.00930)

Figure S2 Rarefaction plot showing curves of OTUs of each sample groups with sequencing depth.

WCP: water from culture ponds; OWASFP: outfall water from the artificial substrate filtering pond; OWAFBFP: outfall water from the artificial substrate floating bed filtering pond; OWBrFP: outfall water from the brush filtering pond; WWSP: water from the storage pond.

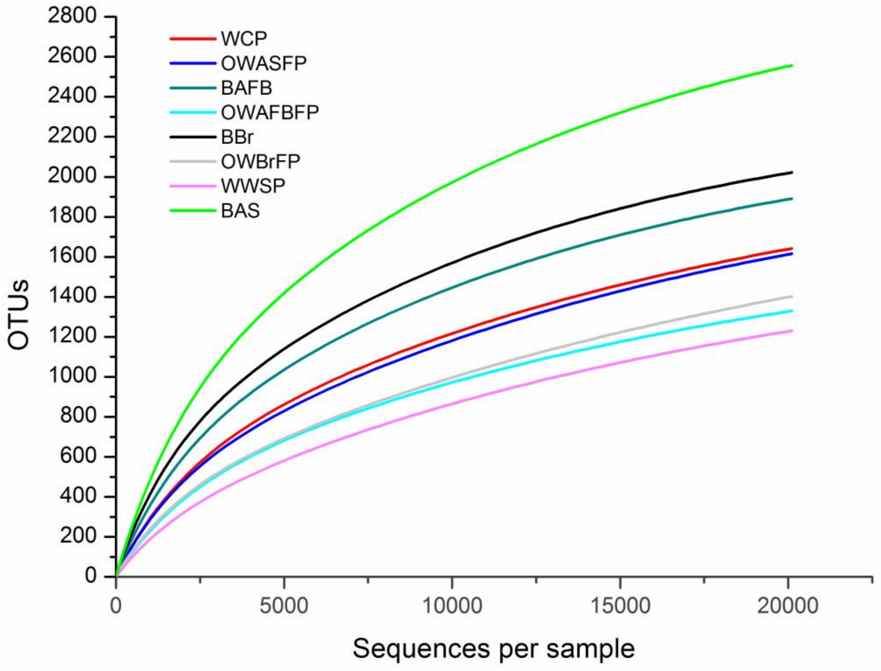

Supplement: Supplementary file 5 [file Image_2.pdf]
